# Articular eminence inclination as a morphological marker for temporomandibular joint disc displacement: a dual-modality imaging analysis

**DOI:** 10.3389/froh.2026.1709454

**Published:** 2026-02-10

**Authors:** Le Thu Huong, Nguyen Thi Thu Phuong, Nguyen Thi Thuy Nga, Vo Hoang Long, Vu Ngoc Bao

**Affiliations:** 1 Hanoi Medical University, Hanoi, Vietnam; 2 Hanoi National Hospital of Odonto Stomatology, Hanoi, Vietnam

**Keywords:** articular eminence, CBCT, disc displacement, mandibular fossa, MRI, temporomandibular joint

## Abstract

**Objective:**

To investigate the association between the morphology of the articular eminence (AE) and mandibular fossa (MF) and the presence of temporomandibular joint (TMJ) disc displacement with reduction (DDwR) using cone-beam computed tomography (CBCT) and magnetic resonance imaging (MRI).

**Methods:**

This retrospective cross-sectional study analyzed 60 TMJs from 30 patients with intra-articular disorders treated at two Vietnamese centers (2022–2024). MRI classified joints as DDwR or normal disc position (No DD). CBCT provided morphometric measurements: AE inclination (top-roof line [TR] and best-fit line [BF] angles), AE height and width, and MF width. Intra-observer reliability was assessed using the intraclass correlation coefficient (ICC). Group differences were tested using t-tests or Mann–Whitney U tests (*p* < 0.05).

**Results:**

MRI identified 50 DDwR joints (83.3%) and 10 No DD joints (16.7%). Measurement reliability was excellent (ICC = 0.86). AE inclination was significantly lower in DDwR joints (TR: 37.85 ± 7.13 ° vs. 46.44 ± 6.41 °, *p* = 0.001; BF: 53.80 ± 6.70 ° vs. 60.64 ± 7.16 °, *p* = 0.003). No significant differences were found for AE height (6.88 ± 1.51 mm vs. 6.91 ± 1.00 mm), AE width (9.37 ± 1.63 mm vs. 9.48 ± 1.54 mm), or MF width (15.24 ± 1.40 mm vs. 15.85 ± 1.00 mm) (all *p* > 0.05).

**Conclusion:**

A flatter AE inclination is significantly associated with DDwR, challenging the traditional view that a steeper eminence predisposes to disc displacement. These findings suggest AE flattening may be a secondary change linked to degenerative remodeling. CBCT-based AE inclination assessment could aid in risk evaluation and early diagnosis of TMJ internal derangement.

## Introduction

The temporomandibular joint (TMJ) is a highly specialized articulation that enables essential functions such as mastication, speech, and swallowing. Structurally, it is formed by the mandibular condyle interacting with the articular eminence and mandibular fossa of the temporal bone, with a fibrocartilaginous disc interposed between them. This disc, together with the surrounding ligaments and musculature, ensures smooth, coordinated mandibular movements. The posterior slope of the articular eminence, in particular, serves as a guiding surface for condylar translation and is critical to maintaining joint stability and overall function within the stomatognathic system (1).

When the structural or functional harmony of the TMJ is disturbed, various temporomandibular disorders (TMDs) can develop. These encompass a spectrum of conditions involving the joint, masticatory muscles, and associated structures. Internal derangement is one of the most frequently encountered subtypes, defined by an abnormal spatial relationship between the articular disc and the condyle–eminence complex (2, 3). In recent decades, imaging techniques such as magnetic resonance imaging (MRI) and cone-beam computed tomography (CBCT) have transformed the evaluation of TMJ pathology. MRI offers detailed visualization of the soft tissue components, particularly disc position and integrity (4), while CBCT provides high-resolution images of the osseous structures, enabling accurate morphometric analysis of the articular eminence and glenoid fossa (5).

Although numerous studies have investigated the relationship between TMJ morphology and internal derangement, the results remain inconsistent. Some authors have reported that a steeper articular eminence inclination may predispose individuals to disc displacement (5); others have suggested the opposite, or found no significant correlation (6). More recent findings indicated that a flatter AE inclination may be associated with disc displacement (7). An alternative hypothesis is that morphological changes, such as articular eminence flattening, are secondary to the progression of joint disease rather than its cause (6). These conflicting interpretations underscore the need for further investigation to clarify whether morphological features of the TMJ contribute to, or result from, internal derangement. A clearer understanding of this relationship could support earlier diagnosis, improve preventive strategies, and inform treatment planning. The present study aims to evaluate the association between the morphology of the articular eminence and glenoid fossa and the occurrence of internal derangement.

## Methods

### Study design and patient selection

This retrospective, cross-sectional investigation explored the relationship between TMJ osseous morphology and the presence of internal derangement. A total of 30 patients, corresponding to 60 TMJs, were selected from individuals receiving treatment for intra-articular disorders at the Hanoi National Hospital of Odonto-Stomatology and the School of Dentistry, Hanoi Medical University, between April 2022 and December 2024. All participants provided written informed consent prior to enrollment, and the protocol was approved by the Institutional Review Board of Hanoi Medical University (Reference: 662/GCN-HĐĐĐNCYSH-ĐHYHN, May 11, 2022).

Eligibility was determined through detailed clinical examination and review of imaging records. Inclusion criteria required a confirmed diagnosis of at least one intra-articular disorder—specifically disc displacement with reduction (DDwR)—as verified by MRI. Clinically, DDwR was diagnosed based on the presence of a reciprocal click during both mouth opening and closing. Joints were categorized as either DDwR or having a normal disc position (No DD).

Exclusion criteria were applied to avoid confounding factors: patients with a history of myogenous disorders (myalgia, myofascial pain), uncontrolled systemic or mental illness, rheumatic disease (e.g., rheumatoid arthritis), craniofacial trauma, or prior TMJ therapy (including stabilization splint use) were excluded. This ensured that the sample reflected intra-articular disorders without interference from unrelated TMD subtypes.

### Imaging protocols and joint classification

Both MRI and CBCT were performed for each patient. MRI examinations were conducted using a 1.5-Tesla system (Siemens Magnetom Avanto, Erlangen, Germany) equipped with a dedicated surface coil. Sagittal and coronal proton density–weighted and T2-weighted sequences were acquired in both closed- and open-mouth positions, with a slice thickness of 3 mm, repetition time (TR) of 2000ms, and echo time (TE) of 25 ms. MRI served as the reference standard for disc position assessment, using sagittal and coronal views in both closed- and open-mouth positions. A diagnosis of DDwR was made when the posterior band of the disc lay anterior to the 11:30 position relative to the condylar head in the closed-mouth view, but repositioned between the condyle and AE in the open-mouth view. A “No DD” classification was assigned when disc position remained normal and stable in both positions. MRI-based disc position classification (DDwR vs. No DD) was independently performed by two calibrated dentomaxillofacial radiologists who were blinded to CBCT measurements and clinical information. Discrepancies between readers were resolved through a consensus reading session, and the final diagnosis was based on the agreed classification. Based on these definitions, 50 joints were assigned to the DDwR group and 10 to the No DD group.

CBCT scans were obtained using a Planmeca Promax® 3D Max unit (Helsinki, Finland) with standardized parameters (96 kV, 5.6 mA, 13 × 13 cm^2^ field of view, 200 μm voxel size). All patients were seated upright with the head in a natural posture and the mouth closed in maximum intercuspation. This protocol yielded high-resolution, multiplanar reconstructions of TMJ bony structures without the superimposition present in conventional radiography.

### CBCT image analysis and morphometric measurements

**Figure 1:**
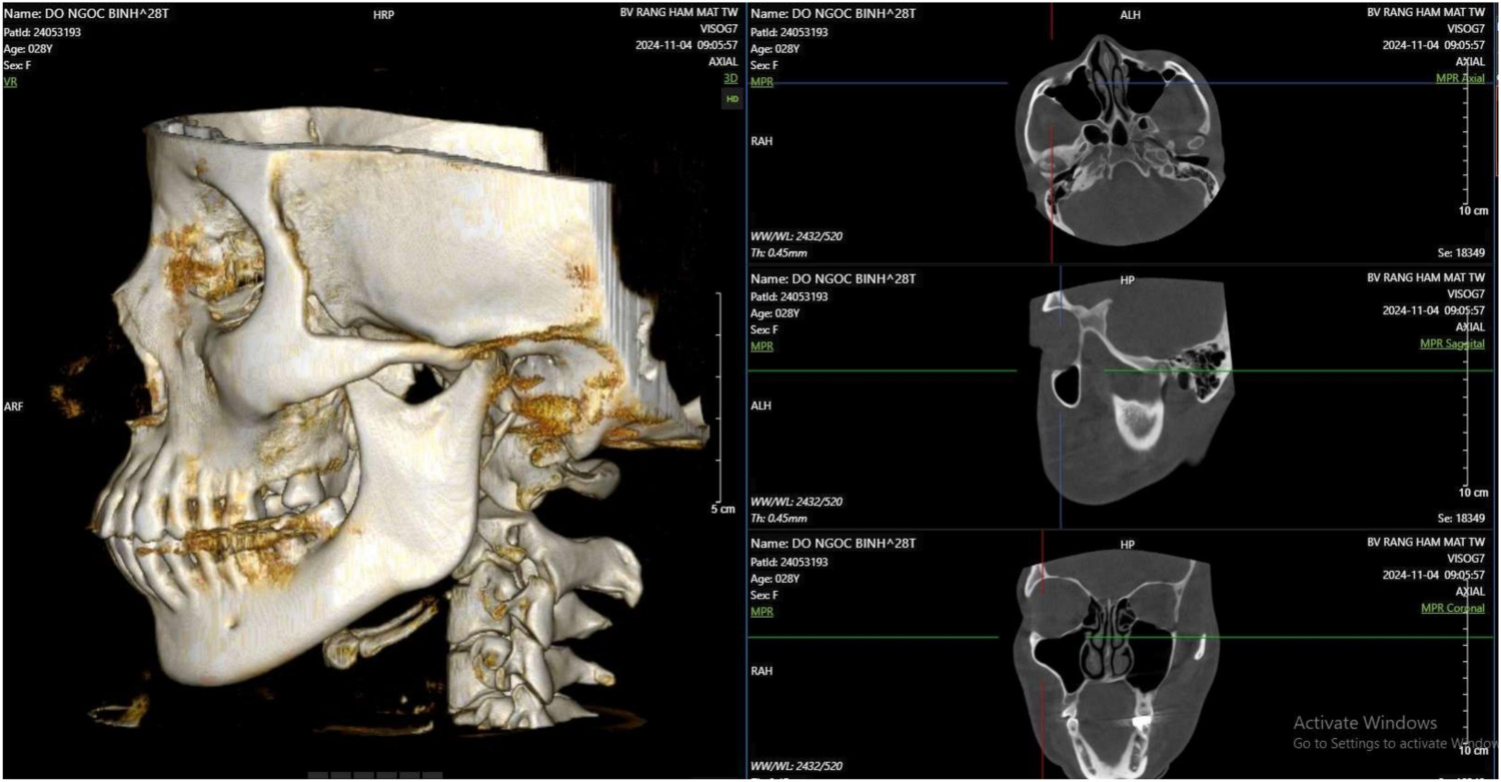
Reorientation of the CBCT axial plane to align with the Frankfort Horizontal (FH) plane.

All CBCT datasets were anonymized and evaluated by a single experienced investigator using Vietrad software (Vietrad Technology JSC, Hanoi, Vietnam, version 3.0*).* The examiner was a dentomaxillofacial radiologist with more than 10 years of clinical and research experience in TMJ imaging. To standardize orientation, the 3D model was rotated until the Frankfort horizontal (FH) plane was parallel to the true horizontal and the axial plane was properly aligned (Figure 1).

**Figure 2:**
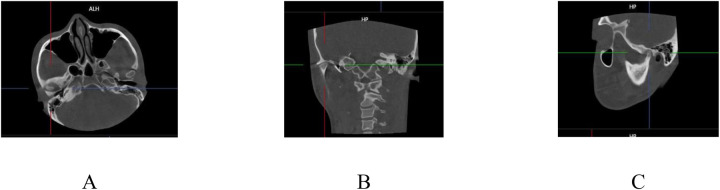
Multiplanar reconstruction of the TMJ for analysis: **(A)** Axial view, **(B)** Central coronal plane, and **(C)** Central sagittal plane.

Paracoronal images were reconstructed along the widest lateral–medial span of the mandibular fossa, and parasagittal slices were generated perpendicular to the fossa's midpoint. Three principal reference planes, axial, central coronal, and central sagittal, were established for subsequent measurements (Figure 2).

**Figure 3:**
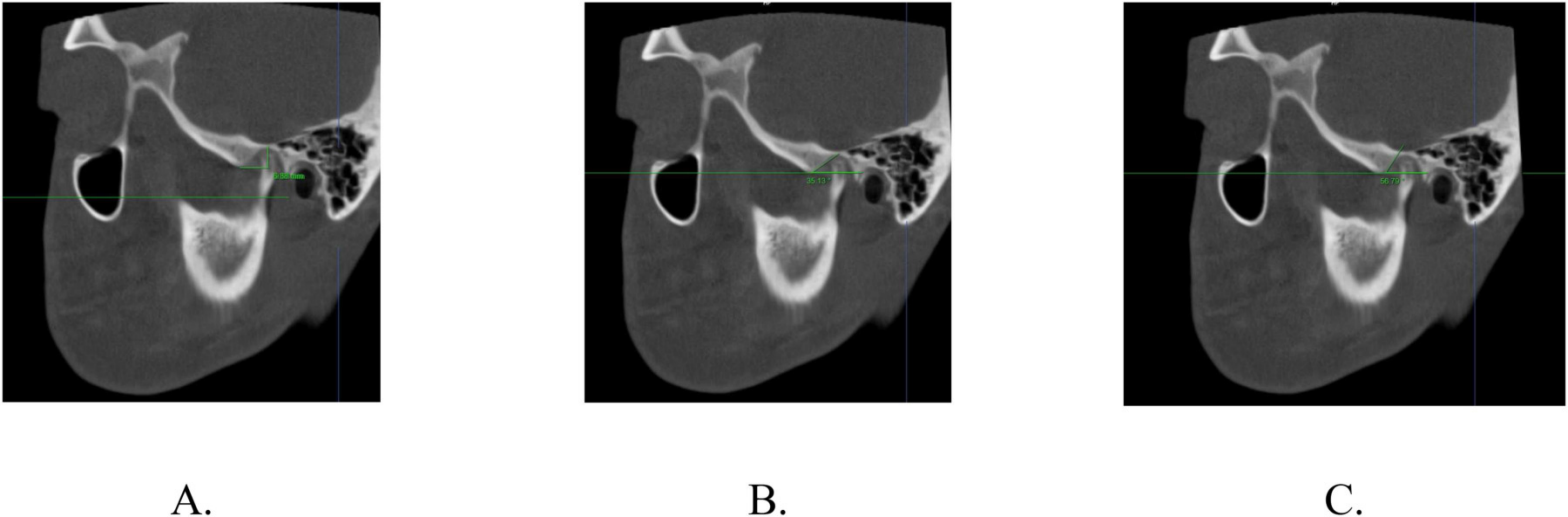
Morphometric measurements of the articular eminence: **(A)** eminence height (EH) and width (EW). EH is the vertical distance from the deepest point of the fossa to the lowest point of the eminence. EW is the horizontal distance from the lowest point of the eminence to a vertical line from the deepest fossa point. **(B)** Top-roof line angle (TR angle) and **(C)** Best-fit line angle (BF angle).

The morphology of the AE was evaluated through specific parameters (Figure 3):

- Eminence Height (EH): Vertical distance from the deepest point of the mandibular fossa to a horizontal reference plane intersecting the lowest point of the AE.
- Eminence Width (EW): Horizontal distance from the lowest point of the AE to a vertical line descending from the deepest fossa point, parallel to FH.
- Top-Roof Line Angle (TR angle): Angle between the horizontal reference plane and a line connecting the lowest point of the AE to the deepest point of the mandibular fossa.
- Best-Fit Line Angle (BF angle): Angle between the tangent to the posterior inclination of the AE and the FH plane.

**Figure 4:**
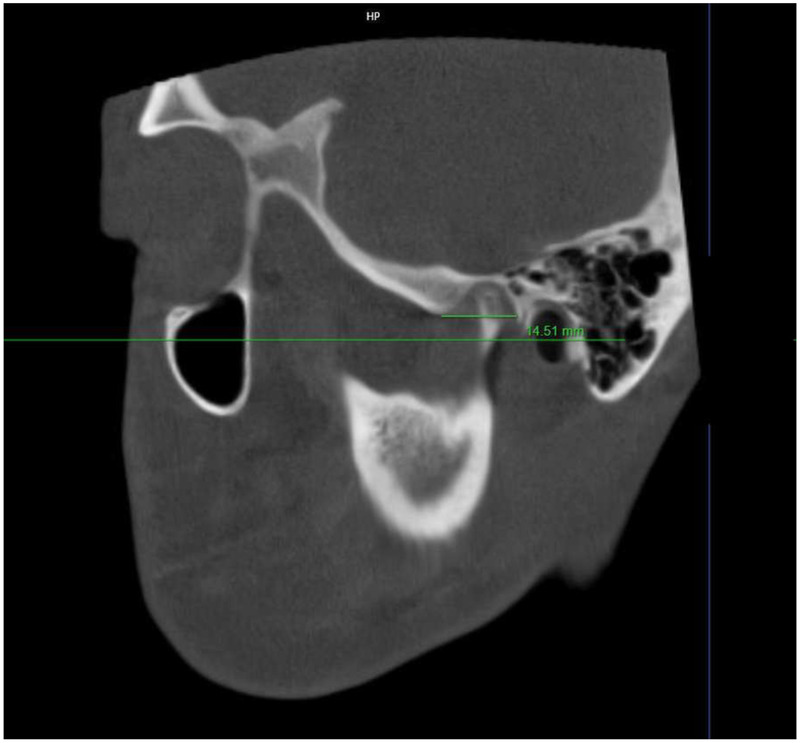
Measurement of mandibular fossa width (FW), defined as the horizontal distance from the lowest point of the articular eminence to the posterior glenoid process.

The fossa width (FW) was determined as the horizontal distance from the lowest point of the AE to the posterior glenoid process (Figure 4).

All measurements were performed three times on separate days, at least one week apart, and averaged for analysis. Intra-observer reliability was quantified using the intraclass correlation coefficient (ICC), which yielded a value of 0.86. According to the criteria proposed by Koo and Li (2016) (8), this represents excellent reliability (ICC ≥ 0.75).

**Figure 5:**
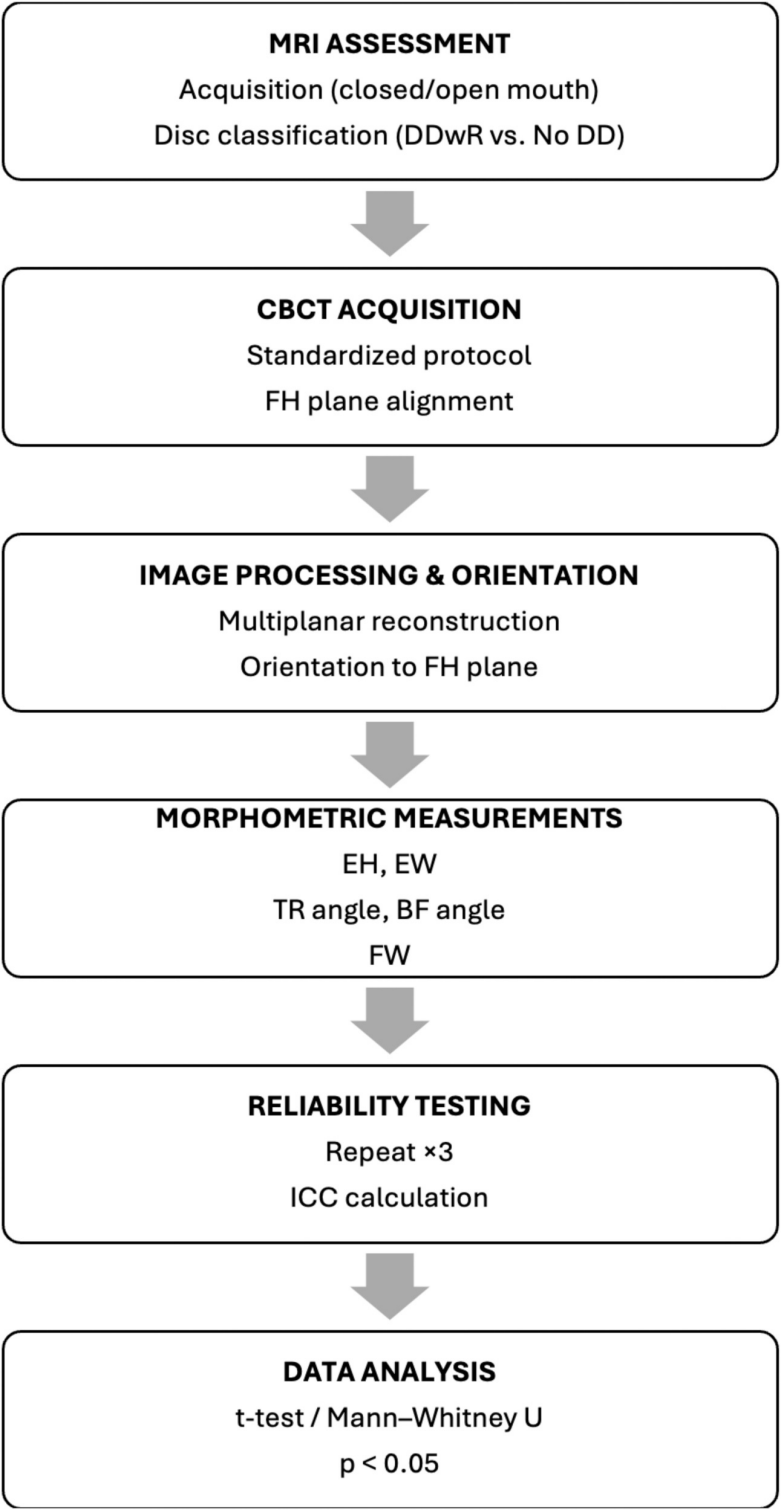
Workflow for MRI and CBCT data evaluation.

Figure 5 illustrates the workflow of clinical imaging data evaluation, from acquisition to statistical analysis.

### Data analysis

Data analysis was performed with SPSS (version 21.0, IBM Corp., Armonk, NY, USA). The Kolmogorov–Smirnov and Shapiro–Wilk tests were conducted to assess the normality of continuous variables. Based on these results, Mann–Whitney U tests were applied to compare the Articular Eminence Inclination and Eminence Width between the DDwR and No DD groups, as these data were not normally distributed. For normally distributed variables, specifically Eminence Height and Mandibular Fossa Width, independent samples t-tests were used to compare the group means. Statistical significance was set at a two-tailed *p* < 0.05.

## Results

A total of 60 TMJs from 30 patients were included in this study. The study population comprised 22 females and 8 males, with a mean age of 28.6 ± 6.4 years (range: 19–42 years). Based on MRI assessments, 50 joints (83.3%) were classified as DDwR, while 10 joints (16.7%) demonstrated a No DD. The intra-observer reliability for all CBCT measurements, assessed by the ICC, was 0.86.

### Articular eminence inclination

As shown in Table 1, the inclination of the AE was significantly lower in the DDwR group compared to the No DD group. When measured by the Top-Roof Line method, the mean angle in the DDwR group was 37.85° ± 7.13°, which was significantly flatter than the 46.44° ± 6.41° observed in the No DD group (*p* = 0.001). Similarly, using the Best-Fit Line method, the mean angle for the DDwR group was 53.80° ± 6.70°, which was also significantly flatter than the 60.64° ± 7.16° in the No DD group (*p* = 0.003).

**Table 1:** The inclination of the AE in CBCT (*n* = 60).

Group	*N*	Mean	SD	Median	Minimum	Maximum	*p*-value
Top roof line ( °)							
DDwR	50	37.85	7.13	38.94	22.48	48.80	0.001^a^*
No DD	10	46.44	6.41	48.40	35.31	52.68	
Best fit line ( °)							
DDwR	50	53.80	6.70	55.11	36.10	69.54	0.003^a^*
No DD	10	60.64	7.16	63.87	43.39	66.62	

^a^ Mann–Whitney *U*-test.

* significant at 0.05; SD: standard deviation.

### Articular eminence dimensions

Table 2 presents the dimensional measurements of the AE. No statistically significant differences were found between the DDwR and No DD groups for either eminence width or height. The mean eminence width for the DDwR group was 9.37 ± 1.63 mm, while the No DD group had a mean width of 9.48 ± 1.54 mm (*p* = 0.812). The mean eminence height in the DDwR group was 6.88 ± 1.51 mm, which was comparable to the 6.91 ± 1.00 mm in the No DD group (*p* = 0.955).

**Table 2:** The dimension of AE in CBCT (*n* = 60).

Group	*N*	Mean	SD	Median	Minimum	Maximum	*p*-value
Eminence width (mm)							
DDwR	50	9.37	1.63	9.39	5.27	13.31	0.812^a^
No DD	10	9.48	1.54	8.73	8.04	12.81	
Eminence height (mm)							
DDwR	50	6.88	1.51	6.67	3.88	9.88	0.955^b^
No DD	10	6.91	1.00	7.23	5.23	8.12	

^a^ Mann–Whitney *U*-test.

^b^ Student's *t*-test; SD: standard deviation.

### Mandibular fossa width

As detailed in Table 3, the mean width of the mandibular fossa was greater in the No DD group (15.85 ± 1.00 mm) compared to the DDwR group (15.24 ± 1.40 mm). This difference, however, was not statistically significant (*p* = 0.194).

**Table 3:** The dimension of mandibular fossa in CBCT (*n* = 60).

Fossa width (mm)	*N*	Mean	SD	Median	Minimum	Maximum	*p*-value
DDwR	50	15.24	1.40	15.27	12.17	18.87	0.194^a^
No DD	10	15.85	1.00	15.81	14.06	17.23	

^a^ Student's *t*-test; SD: standard deviation.

## Discussion

The TMJ stands as a marvel of biological engineering, and disruptions to its biomechanics, particularly internal derangement, are a frequent cause of craniomandibular dysfunction. The osseous morphology of the TMJ, encompassing the AE and the mandibular fossa, is widely believed to play a crucial role in joint function and pathology. However, the precise nature of this relationship remains a subject of ongoing debate and conflicting evidence in the literature. Our study aimed to clarify this association by meticulously measuring these structures in joints with and without DDwR, utilizing a rigorous, dual-modality imaging approach. The combination of MRI for definitive disc position classification and CBCT for high-resolution osseous analysis represents a significant methodological strength, mitigating the diagnostic ambiguities that have often limited the conclusiveness of prior research.

Our most compelling finding is the statistically significant association between a flatter Articular Eminence Inclination and the presence of DDwR. The DDwR group consistently exhibited significantly lower mean angles for both the “top-roof line” and “best-fit line” methods compared to the group with a No DD. This result challenges the traditional biomechanical theory which posits that a steeper eminence predisposes an individual to disc displacement by increasing the likelihood of disc luxation during movement. Instead, our findings align with the perspective of other studies, such as that by Kim et al. (2022) (7), which also observed a gentler posterior eminence slope in joints with dislocation. This suggests a potential alternative biomechanical pathway: a less-inclined posterior slope might provide inadequate guiding force or insufficient structural support for the condyle-disc complex, thereby compromising joint stability and increasing susceptibility to disc displacement. The biomechanical interplay between Articular Eminence Inclination and muscle function is also a critical consideration. A steeper Articular Eminence Inclination may require a greater distalizing force from the masseter and temporalis muscles to maintain joint stability, as proposed in some theories (9). Conversely, a flatter eminence might alter these forces in a way that contributes to anterior disc shifting over time.

From a clinical standpoint, the observation that joints with DDwR displayed flatter eminences highlights potential diagnostic and preventive implications. CBCT-derived Articular Eminence Inclination measurements could be applied as an adjunctive tool in screening patients at risk of internal derangement. In particular, individuals presenting with joint sounds such as clicking but without significant symptoms might benefit from CBCT evaluation to detect subtle morphological predispositions. Such an approach could support earlier diagnosis, facilitate preventive counseling, and guide closer follow-up, especially in settings where MRI is not available or contraindicated. However, given the relatively small and imbalanced control group and the cross-sectional design of the present study, we did not attempt to define a specific cut-off value for Articular Eminence Inclination for clinical screening, as any threshold derived under these conditions would be prone to instability and limited generalizability. Accordingly, the observed difference in inclination should be interpreted as an anatomical association rather than a diagnostic criterion. Future validation studies are warranted to determine whether AE flattening can serve as a reliable predictive marker in asymptomatic or subclinical populations, ideally through receiver operating characteristic analysis and external validation in larger, balanced cohorts.

Furthermore, our results lend strong support to the hypothesis that the flattening of the AE may be a secondary consequence of the degenerative changes associated with internal derangement, rather than a primary etiological factor. The chronic, repetitive microtrauma and altered loading patterns inherent to a displaced disc can induce adaptive or degenerative bone remodeling. This process, as suggested by studies from Kurita et al. (2000) (6) and Yamada et al. (2004) (10), often manifests as a progressive decrease in eminence steepness as the condition advances from early internal derangement to more severe forms. This critical distinction in the temporal relationship between morphology and pathology positions the osseous structure not as a simple predictor of onset, but rather as a valuable marker reflecting the history and progression of joint dysfunction.

Beyond this hypothesis, alternative mechanisms should also be considered. Altered loading from the masticatory muscles may exert abnormal mechanical stresses on the joint surface, modifying the direction and magnitude of forces applied to the eminence. Such changes could stimulate adaptive bone remodeling, consistent with Wolff's law, resulting in a gradual flattening of the AE. In addition, inflammatory processes associated with disc displacement may accelerate subchondral bone resorption and remodeling, further contributing to morphological alterations. These pathways highlight the multifactorial nature of AE flattening, involving not only degenerative progression but also muscle dynamics and local inflammatory responses (11, 12).

In contrast to our findings on Articular Eminence Inclination, our study found no statistically significant differences in the height or width of the AE, nor in the width of the mandibular fossa, between the two groups. This outcome is consistent with studies by Sravya Vemareddy et al. (2019) (13) and Al Rawi et al. (2017) (14), who also found no significant height differences, suggesting that the overall dimensions of the eminence and fossa may not be as critical a determinant of disc position as the specific angle of the gliding path. This implies that the functional geometry of the joint surface, as captured by Articular Eminence Inclination, holds more profound biomechanical significance for the disc-condyle relationship than its gross size. Our results regarding fossa width do diverge from those of Paknahad et al. (2016) (15), who reported a wider fossa in TMD patients. This discrepancy might be attributable to differences in patient selection and diagnostic criteria. Our study focused exclusively on patients with MRI-confirmed DDwR, whereas other studies may have included a broader spectrum of TMDs, potentially introducing confounding variables.

The morphological patterns we observed, including smaller mean dimensions for all measured structures in the DDwR group, though not statistically significant, suggest a structural interdependence. This necessitates a cautious interpretation and underscores the need for larger-scale studies to confirm these subtle associations. Given these findings, it is reasonable to suggest that CBCT, despite its limitations in soft tissue visualization, can serve as a valuable and cost-effective screening tool. By identifying specific morphological markers like a flatter Articular Eminence Inclination, CBCT can help clinicians identify joints with a higher likelihood of internal derangement, particularly in cases where MRI is not a feasible option due to cost, accessibility, or patient contraindications (e.g., claustrophobia, metal implants) (16).

While our study provides valuable insights, it is not without limitations. The relatively small sample size, particularly in the No DD group (only 10 joints), may have limited the statistical power to detect subtle but clinically relevant differences in eminence and fossa dimensions. This imbalance largely reflects the clinical referral pattern of patients undergoing combined MRI and CBCT assessment, in which joints with suspected disc displacement are more frequently indicated for advanced imaging. Moreover, the imbalance in group sizes raises the possibility of reduced robustness in comparative analysis, especially for secondary morphometric parameters with smaller effect sizes, and may limit the generalizability of the findings. In addition, although MRI-based disc position assessment was independently performed by experienced radiologists with final diagnoses reached by consensus, inter-rater reliability was not quantitatively assessed using Cohen's kappa, which may limit the formal evaluation of diagnostic reproducibility. The cross-sectional design of this study also precludes us from establishing a definitive cause-and-effect relationship, leaving open the question of whether a flatter eminence is a precursor to or a consequence of disc displacement. Therefore, future research should employ a larger and more balanced cohort, particularly with an expanded control (No DD) group, and, ideally, a longitudinal design to track morphological changes over time. Prospective studies should also incorporate formal inter-rater reliability analysis to further strengthen the objectivity of MRI-based joint classification. Further investigation into the biomechanical role of Articular Eminence Inclination, perhaps through dynamic imaging techniques or computational modeling, could provide a more detailed understanding of its influence on joint stability. By refining our understanding of these structural associations, we can improve early risk assessment and inform more targeted, personalized treatment strategies.

## Conclusion

This study reveals a significant association between a flatter articular eminence inclination and TMJ DDwR. Joints with DDwR consistently demonstrated a less-inclined articular eminence. This finding challenges the traditional belief that a steeper eminence is a primary risk factor for internal derangement, suggesting instead that flattening may be a secondary consequence of degenerative remodeling. The study emphasizes the importance of analyzing articular eminence inclination. This analysis can provide critical insights for diagnosing disc displacement and deepening our knowledge of TMJ biomechanics.

## Data Availability

The raw data supporting the conclusions of this article will be made available by the authors, without undue reservation.
